# The Growth Performance, Nutrient Digestibility, Gut Bacteria and Bone Strength of Broilers Offered Alternative, Sustainable Diets Varying in Nutrient Specification and Phytase Dose

**DOI:** 10.3390/ani12131669

**Published:** 2022-06-29

**Authors:** Christina C. Mulvenna, Ursula M. McCormack, Elizabeth Magowan, John McKillen, Mike R. Bedford, Carrie L. Walk, Michael Oster, Henry Reyer, Klaus Wimmers, Dario A. Fornara, M. Elizabeth E. Ball

**Affiliations:** 1 Agri-Food & Biosciences Institute (AFBI), Newforge Lane, Belfast BT9 5PX, UK; ursula.mccormack@dsm.com (U.M.M.); elizabeth.magowan@afbini.gov.uk (E.M.); john.mckillen@daera-ni.gov.uk (J.M.); d.fornara@davines.it (D.A.F.); elizabeth.ball@afbini.gov.uk (M.E.E.B.); 2 DSM Nutritional Products France, Centre De Recherche En Nutrition Animale, 68305 Saint-Louis, France; 3 AB Vista, Marlborough, Wiltshire SN8 4AN, UK; mike.bedford@abvista.com (M.R.B.); carrie.walk@dsm.com (C.L.W.); 4 DSM Nutritional Products, Delves Road, Heanor, Derbyshire DE75 7SG, UK; 5 Institute of Genome Biology, Research Institute for Farm Animal Biology (FBN), 18196 Dummerstorf, Germany; oster@fbn-dummerstorf.de (M.O.); reyer@fbn-dummerstorf.de (H.R.); wimmers@fbn-dummerstorf.de (K.W.)

**Keywords:** broiler, performance, digestibility, phytase, phosphorus

## Abstract

**Simple Summary:**

Phytase has been routinely included in poultry diets for over 20 years with the benefits to bird growth performance, nutrient digestibility and bone parameters widely documented. However, with the costs of feed increasing, particularly of imported soyabean meal and pressure to reduce the environmental footprint of production, the need for alternative sustainable local protein sources is ever important. This work found that phytase superdosing to rapeseed meal (RSM) and maize dried distiller grains with solubles (DDGS) diets can improve performance, increase digestibility and reduce P excretion by 15% compared to birds offered the diet without exogenous phytase.

**Abstract:**

This study assessed the use of locally sourced sustainable feed ingredients, rapeseed meal (RSM) and maize dried distiller grains with solubles (DDGS) in diets over traditional ingredients on the growth performance, bone strength and nutrient digestibility of broilers. This work also investigated the effects of supplementing exogenous phytase in two doses (500 vs. 1500 FTU/kg). Using male Ross 308 chicks (*n* = 320) assigned to receive one of four experimental diets: (1) Positive control diet 1 (PC1), a wheat, soya-based diet + 500 FTU/kg phytase. (2) Positive control diet 2, RSM/DDGS diet + 500 FTU/kg phytase (PC2). (3) Negative control (NC) reduced nutrient RSM/DDGS diet, no phytase. (4) The NC diet plus 1500 FTU/kg phytase (NC+). PC1 birds displayed higher feed intake and body weight gain consistently throughout the trial (*p* < 0.001) as well as increased body weight by 28 d and 42 d (*p* < 0.001). Whole-body dual emission X-ray absorptiometry (DXA) analysis revealed PC1 birds also had higher bone mineral density (BMD), bone mineral content (BMC), total bone mass, total lean mass and total fat mass than birds offered other treatments (*p* < 0.01). Diet had no significant effect on bone strength. Phytase superdosing improved the digestibility of dry matter (DM), neutral detergent fibre (NDF), gross energy (GE), calcium (Ca), potassium (K) and magnesium (Mg) compared to birds in other treatment groups. The phytase superdose also improved performance in comparison to birds offered the NC diet. Phytase superdosing increased the IP6 and IP5 degradation and increased the ileal inositol concentration of the birds. N excretion was lower for birds offered the traditional wheat–soya diet and highest for those offered the high-specification RSM/DDGS diet with a commercial dose of phytase. The addition of a phytase superdose to the negative control diet (NC+) reduced P excretion of birds by 15% compared to birds offered NC.

## 1. Introduction

Phosphorus (P) is an essential nutrient for poultry and is utilised in a number of biological processes, including energy metabolism, muscle growth, bone development and egg production. However, around 60 to 80% of the total P present in animal feed is bound to phytate (myo-inositol hexaphosphate, IP6). Poultry lack sufficient gastrointestinal quantities of the enzyme phytase required for the degradation of the phytate molecule into lower phosphor-ester forms (IP2-5 and inositol). As a result, phytate cannot be digested and leaves large amounts of P inaccessible and also reduces access to other important nutrients, amino acids and energy [1,2]. Therefore, diets are often supplemented with inorganic-P in order to meet bird requirements. However, this approach is dependent on finite inorganic-P sources, increases diet costs and can result in significantly high P excretion which has severe environmental implications [3]. While plant-based feed ingredients contain levels of endogenous phytase which can improve the P utilisation of the diet, this is often destroyed during the diet manufacturing process, e.g., heat-treating. Therefore, exogenous enzymes with the ability to withstand the diet-manufacturing process have been developed. The addition of exogenous phytase at conventional levels (<500 FTU/kg) into broiler diets has increased over the past 20 years and is one of the most routinely used feed additives in poultry diets. This is due to the well-documented improvements to the digestibility of P, energy and other nutrients [1,2,4,5,6,7,8]. In recent work [9], the addition of phytase even in low doses of 250 FTU/kg yielded a 6.6% and 9.5% increase in P digestibility by day 14 and 28, respectively, and improved the digestibility of all amino acids in birds by 28 days of age. This increased access and improved digestibility of available nutrients, energy and amino acids explains the consistent evidence that the addition of phytase to broiler diets leads to improvements in growth performance (i.e., increased weight gain and improved feed efficiency) and bone parameters such as increased strength and bone ash [2,4,9,10,11,12,13].

As mentioned, excess P in poultry manure nearby to water sources can result in eutrophication and other forms of pollution [14]. Therefore, an increase in P utilization as a result of phytase inclusion can reduce the P lost in manure [14,15,16]. Work has shown that reductions in P excretion by 30–40% can be achieved through the addition of 500 FTU/kg phytase [17,18]. Therefore, the addition of phytase does not only have benefits in terms of production and economics but is also of environmental importance.

The addition of phytase at levels of 500 FTU/kg is considered common practice. The idea of “superdosing” defined as ‘using levels of phytase in greater quantities than required to fulfil P requirements’ [19] is gaining more attention due to rising ingredient costs. Scholey et al. [20] investigated the effects of phytase supplementation in doses from 500 to 1000 FTU/kg in diets low in inorganic-P and reported that birds offered the control diet with higher levels of inorganic-P (16.20, 10.90, 9.40 and 6.10 g/kg—Starter, Grower 1, Grower 2 and Finisher phase, respectively) outperformed broilers offered the low inorganic-P diets supplemented with 500 and 750 FTU/kg. The authors suggested that this may be due to an incomplete dephosphorylation of the inositol ring of phytate, therefore suggesting that doses of 1000 FTU/kg or higher may be required in the grower period to allow complete destruction of phytate-P and access to nutrients. In other work, increasing the amount of additional phytase to broiler diets from 0, 500, 1000 and 2000 to 4000 FTU/kg resulted in a quadratic increase in body weight, feed intake, tibia ash and a linear increase in the digestibility of energy and all amino acids. Furthermore, increasing phytase from 0 to 4000 FTU/kg phytase improved the ileal digestibility of P and Ca by 88 and 18%, respectively [21], and more evidence shows that phytase superdosing improves bone development [22], with Walk et al. [23] reporting increased growth performance, increased tibia ash and as well as reduced phytate concentration in the gizzard of birds offered the higher levels of phytase (1500 FTU/kg). These findings have been reiterated by the work of others [6,8,12,22,23,24,25] all of which have reported additional beneficial effects to superdosing. There is also evidence to suggest that phytase superdosing can alter the morphology of the intestine, e.g., increased villi height and villi height to crypt depth ratio (VH:CD) [26,27] and the gut microbiota by increasing beneficial bacteria, e.g., *Lactobacillus*, while reducing harmful bacteria [27,28], ultimately improving broiler gut health and facilitating the improved absorption of nutrients.

While it is clear that superdosing phytase into poultry diets is not a new concept, many of the aforementioned studies have focused on the effects of phytase on traditional diets comprised of cereals and soyabean meal. However, soyabean meal is an expensive component and must be imported, thus increasing the environmental footprint of production. Current efforts to switch to home grown alternatives such as rapeseed meal (RSM) and dried distiller grains with solubles (DDGS) to reduce the use of soyabean meal have been shown to be successful in maintaining bird performance [29]. However, the effects of phytase addition, if any, or the levels of dosing required to these alternative, more sustainable diets are yet to be fully investigated. The objective of this work was to evaluate the use of locally sourced RSM and maize DDGS diets as sustainable alternatives to traditional soya broiler diets. We aimed to investigate the effects of phytase inclusion at two different doses (500 and 1500 FTU/kg) to these sustainable diets and whether this would enhance broiler performance, nutrient digestibility, gut bacteria and bone strength to levels similar to or exceeding birds offered the traditional diets. 

## 2. Materials and Methods

### 2.1. Ethical Declaration

All procedures conducted within this study were approved by the Animal Welfare Ethical Review Body and in accordance with the Animals Scientific Act 1986, [30] at the Agri-Food Bioscience Institute (AFBI). Two trials were conducted as part of this study, a performance trial and a digestibility trial.

### 2.2. Diet Treatments

Details of dietary ingredients for all diets are presented in Table 1. The positive control diet (PC1) consisted of a wheat, soya-based diet with an exogenous phytase dose at commercial levels (500 FTU, 0.01%, Quantum^®^ Blue, AB Vista, Wiltshire, UK). A second positive control diet (PC2) was an alternative sustainable diet containing RSM, maize DDGS and commercial levels of exogenous phytase (500 FTU, 0.01%, Quantum^®^ Blue). The negative control treatment (NC) was a sustainable diet containing RSM, maize DDGS with no exogenous phytase, reduced in energy (3% and 2% in starter/grower and finisher diets, respectively), P (2 g/kg), calcium (Ca, 2.4 g/kg) and essential amino acids (reductions of 0.3 g/kg lysine, 0.6 g/kg methionine, 0.6 g/kg threonine and 0.3 g/kg tryptophan) compared to positive control diets. A further experimental treatment (NC+) was obtained by supplementing the NC diet with a phytase superdose (1500 FTU, 0.03%, Quantum^®^ Blue). Each diet was provided in pelleted form ad libitum to birds on each trial.

### 2.3. Performance Trial 

Male Ross 308 chicks (*n* = 320) of similar weight (40.15 ± 0.28 g) were randomly assigned to one of 32 solid floor pens (10 birds/pen). Each pen was 1.5 × 0.9 m and 2.5 kg of wood shavings were provided. Initial room temperature was 32 °C, which was reduced by 0.5 °C per day until it reached 22 °C, after which it was maintained at this temperature. Birds were exposed to a light: dark cycle of 18:6 hr. In the pens, birds were provided with one of the four experimental treatment diets (80 birds/treatment, 8 pens/treatment). From day 0 to 28, chicks were offered starter/grower formulations of the diets ad libitum. On day 7 of the trial, two birds per pen (*n* = 64) were selected and transferred to metabolism cages for the digestibility trial, while the remaining birds (*n* = 256) continued on the performance trial. Finisher formulations of diets were provided ad libitum from day 29 to day 42. Birds and feed were weighed every seven days to determine body weight (BW), feed intake (FI) and body weight gain (BWG) for starter/grower (0–28 d), finisher (29–42 d) and overall (0–42 d) phases. From this, feed conversion ratio (FCR) was calculated as the ratio of feed intake per pen to the average weekly weight gain per pen for the same time phases. Body weight gain was also used to calculate the days of production which would be required for birds in each group to reach a target weight of 2.2 kg. On day 42, all birds remaining on the performance trial (*n* = 256) were euthanized and two from each pen (*n* = 64) of average pen weight were individually scanned using a dual emission X-ray absorptiometry (DXA) scanner (Stratos DR Bone Densitometer, DMS, Mauguio, France). Each scan was analysed using the software 3D DXA, Medix DR to determine body and bone composition, i.e., total bone mass, total fat mass, total lean mass, bone mineral content (BMC) and density (BMD). The right tibia of these birds (*n* = 64) was then dissected for bone strength analysis. After dissection and cleaning, bones were frozen until required for measurement. Prior to measurements, bones were thawed at room temperature overnight. Each tibia bone was then weighed (g), bone length and diameter were measured using digital callipers (mm). The DXA scanner was then used to scan each tibia individually, the region of interest was selected and contained both trabecular and cortical bone. The scans were analysed using the software 3D DXA, Medix DR for measurements of bone mineral content (BMC; g) and bone mineral density (BMD; g/cm^2^). A breaking strength test was conducted on each bone to determine max strength (kg). This involved subjecting the bones to a specific load using a 3-point adjustable bending jig (5KN Flexure fixture, INSTRON^®^, Norwood, MA, USA) with a 30 mm anvil, according to the method of Crenshaw et al. [31]. The load cell was 100 kg and a crosshead speed of 25 mm/min was applied. The force of an attached anvil measuring 50 mm in length and 10 mm wide was applied to the midpoint of the same facial plane of each bone supported by two supports 30 mm apart until the bone failed (determined automatically by the machine). 

### 2.4. Digestibility Trial

The two birds from each pen removed on day 7 (*n* = 64) of the performance trial were transferred to individual metabolism cages (31 × 22.5 × 30 cm^3^). Initial room temperature was 33 °C, which was reduced by 1 °C every two days until it reached 24 °C. Within the metabolism cages, birds were exposed to a light: dark cycle of 18:6 hr. Birds continued on their previously assigned starter/grower formulations and were offered feed and water ad libitum from 7 to 28 d. Excreta were collected twice daily over the 14–21 d period and stored at 4 °C until samples were dried at 80 °C for 48 h and milled for subsequent analysis of dry matter (DM), ash, neutral detergent fibre (NDF), gross energy (GE), lipid, P, Ca, potassium (K) and magnesium (Mg). It was planned to keep the birds in cages until excreta corresponding to the finisher diet were collected but this was not possible. Instead, a second batch of birds (*n* = 32) were kept in group pens and offered one of the four finisher diets to obtain ileal digesta at slaughter (8 birds/treatment). At 42 d, birds were humanely killed by an overdose of isoflurane and confirmed by dislocation of the spinal cord. Birds were then dissected and samples of ileal digesta were taken and frozen until dried at −80 °C for 48 h and subsequently analysed for DM, ash and TiO_2_. In addition, sampling included jejunal digesta which has been collected proximal to Meckel’s diverticulum. Jejunal samples were opened with scissors and the digesta collected. Digesta samples were frozen on dry ice and stored at −80 °C until further analysis.

#### 2.4.1. Chemical Analysis 

The dried samples of the diets, excreta and ileal digesta were used to determine the digestibility of DM, ash, GE, NDF, lipid, N, Ca, P, K and Mg. Dry matter was determined by oven-drying (Gallenkamp, London, UK) at 100 °C for 24 h [32]. Ash content was determined by ashing samples in a muffle furnace (Vecstar, Chesterfield, UK) at 550 °C for approximately 10 h. The GE content was measured using a 6300/6400 bomb calorimeter system (Parr© Instruments, Moline, IL, USA) and NDF was determined using the Fibertec system (Tecator Ltd., Bristol, UK) according to the method of Van Soest [33]. Oil B content was determined by acid hydrolysis (3 M-HCl), followed by solvent extraction with petroleum ether (40 to 60 °C) in a SoxROC Extraction Unit (OPSIS^®^, Furulund, Sweden). Nitrogen content was analysed by the macro-Kjeldahl technique using a 2400/2460 Tecator Kjeltec Auto Sampler System (FOSS, Hillerød, Denmark). For the determination of P, Ca, Mg, and K content, samples were ashed at 500 °C overnight. P content was determined using a Perkin Elmer Flow Injection Analyzer (model FIAS 300, Waltham, MA, USA), with the measurement of the resultant molybdenum blue complex conducted at 700 nm via a Perkin Elmer Lambda 2 Spectrophotometer. Ca, Mg and K contents were determined by being analysed by inductively coupled plasma–optical emission spectroscopy (ICP-OES) following an aqua regia digestion step according to AOAC 985.01. Quantum Blue phytase activity was analysed specifically in diets by the ELISA method using Quantiplate Kits for Quantum Blue (Enzyme Services and Consultancy Ltd., Ystrad Mynach, UK) and total phytase activity was measured in the same laboratory using the Standard Analytical Method 020 (AB Vista), whereas phytate was analysed by near-infrared spectroscopy (NIRS^TM^, Model DS2500, FOSS, Hillerød, Denmark). The spectrometer used covers the range 400–2500 nm 0.5 nm resolution, but for the development of the NIR Phytate Calibration only the region 1100–2500 nm was considered. Titanium dioxide (TiO_2_) was added to all diets during both starter/grower and finisher stages as an indigestible marker to determine nutrient digestibility. TiO_2_ content was measured according to the procedure by Leone [34] and modified by Peddie et al. [35]. Amino acid content was determined by ion-exchange chromatography and conducted by Sciantec Ltd. according to methods of the European Commission [36]. Total N intake (g) and N retention (g) were calculated for the starter/grower (0–28 d), finisher (29–42 d) and overall (0–42 d) phases. The calculations used were:

N intake (g) = DFI (g) × CP (g)/6.25(1)

where CP is crude protein on a fresh-matter basis and:

N retention (g) = DLWG (g) × 0.18/6.25(2)

where DLWG is daily live weight gain.

To calculate each of these on a g/day basis, this was then divided by the days in each phase. 

Levels of N and P excretion were also calculated for the starter/grower (0–28 d), finisher (29–42 d) and overall (0–42 d) phases as:

X excretion (g) = X intake − X retention(3)

where X represents N or P.

N and P excretion were also calculated on a g/kg body weight gain basis for 0–28 d, 29–42 d and 0–42 d phases, and using the predicted days to reach target weight, the estimated total N and P excretion was calculated as: days to reach 2.2 kg target weight × N or P excretion (g/day).

Inositol and inositol phosphates (IP6-IP2) were extracted from dry feed and freeze-dried digesta and processed for HPLC according to Lu et al. [37]. Inositol phosphates were analysed by anion-exchange HPLC on a 3 mm × 250 mm CarboPac PA200 column, fitted with a guard column 3 mm × 50 mm of the same material. Inositol phosphates were eluted at a flow rate of 0.4 mL/min with a gradient of methanesulfonic and detected by the post-column addition of ferric nitrate in perchloric acid, measured at 290 nm [38]. The HPLC machine (Jasco HPLC, Tokyo, Japan) comprised an AS-4150 autosampler, a PU-4080i inert pump, a PU-4085i semi micro pump, a UV-4070 UV/Vis detector and CO-4061 column oven. Individual peaks of inositol phosphates were integrated with Jasco ChromNav v2 software. Integrated peak areas of different isomers classified by number of phosphates, e.g., IP2, were summed and reported as ‘IP2, IP3, IP4, IP5 and IP6′. Calculations assumed equal detector response for the different classes of inositol phosphate and were calibrated against standards of InsP6 dodecasodium salt (Merck Millipore—Calbiochem Cat: 407125-25 mg, Lot: 2663470, Burlington, MA, USA). A set of standards for identification of the different classes was obtained by acid hydrolysis of phytate (Sigma P8810, St. Louis, MO, USA) [39]. 

The apparent digestibility of DM, GE, ash, Oil B, NDF, Ca, K, Mg, N and P were calculated as in Schiavone et al. [40] using the following formula:

AD (%) = 100 − [100 × (%Ash_diet_/%Ash_excreta_) × (%X_excreta_/%X_diet_)] (4)

where X represents DM, GE, ash, Oil B, NDF, Ca, K, Mg, N and P.

Using the results of GE digestibility from the starter/grower birds, we estimated the apparent metabolizable energy (AME) for each of the diets by multiplying the analysed GE available (fresh basis) by the GE digestibility for each of the treatments.

#### 2.4.2. 16S rRNA Profiling 

Jejunal digesta samples were subjected to DNA extraction employing the DNeasy PowerLyzer PowerSoil Kit (QIAGEN, Hilden, Germany). The extraction procedure included additional incubation steps for 10 min at 70 °C and 10 min at 95 °C and bead beating with a Precellys 24 homogenizer (PEQLab Biotechnology GmbH, Darmstadt, Germany). Amplicon PCR for the 16S rRNA V4 region was performed in duplicate with 515′F and 806R primers [41,42]. Amplicons were purified and normalized with a SequalPrep normalization plate (Invitrogen, Carlsbad, CA, USA). Generated sequencing reads from a HiSeq2500 instrument (Illumina, San Diego, CA, USA) were processed in the mothur software (version 1.44.1). This comprised the alignment of reads to the Silva reference database (release 138) and the generation of operational taxonomic units (OTU) at a sequence identity of 97%. OTUs assigned to unclassified genera were excluded from further analysis. Subsampling was performed considering the sample with the lowest number of reads (140,732 reads).

#### 2.4.3. Statistical Analysis 

One-way analysis of variance (ANOVA) was used to analyse the data using Genstat (19th Edition). For performance parameters, pen was taken as the experimental unit and for bone strength parameters, BMD, BMC and nutrient digestibility parameters, bird was taken as the experimental unit. Bone weight and final weight (day 42) were included as a covariates for bone parameters. If a significant difference was found (*p* < 0.05), a Fisher’s least significant difference test was used to test the pairwise differences between the treatment means. Differences in microbial communities were analysed at genus level using the Kruskal–Wallis H-test implemented in statistical analysis of taxonomic and functional profiles (STAMP, version 2.1.3) and *p* values were adjusted to multiple testing according to the Benjamini–Hochberg approach.

## 3. Results

Details of the analysed diet contents are presented in Table 2. The CP content of each diet was similar to the formulated levels. Where diets NC and NC+ were formulated to reduce Ca content, the analysis revealed that Ca was higher than was formulated in NC (by 1.2 and 2.7 g/kg) and NC+ (by 2.7 and 1.4 g/kg) in both the starter/grower and finisher diets, respectively, resulting in higher-than-formulated Ca:avP ratios. P content was lower than that formulated in the positive control diets in both the starter/grower and finisher diets; this was also the case for nPP. However, the analysed nPP was similar to the formulated nPP in the NC and NC+ diets. Overall, the analysed content of lysine (Lys), methionine (Met), threonine (Thr) and tryptophan (Trp) was similar to the formulated amounts in the PC1 and PC2 diets for both starter/grower and finisher stages. However, the analysed levels of Lys, Met, Thr and Trp in the NC and NC+ diets were higher than formulated. Analysis of diets revealed that Quantum Blue phytase levels were only slightly higher than those planned (i.e., 500 FTU/kg) in the positive control diets and levels in the superdose diet were lower than the planned 1500 FTU/kg (by 110 and 330 FTU/kg, respectively). Diets contained levels of endogenous phytase in both starter/grower and finisher formulations (Table 2); as a result, the total phytase activity was greater across all diets than anticipated from formulated levels. The starter/grower NC diet contained 550 FTU/kg and the finisher NC diet contained 365 FTU/kg endogenous phytase. 

### 3.1. Growth Performance

The results of bird growth performance are reported in Table 3. The feed intake (FI) of birds in PC1 was significantly higher (*p* < 0.001) than birds in all other treatment groups during the starter/grower phase and overall phase. During the finisher phase, the FI of birds offered PC1 was 11% greater (*p* = 0.036) than birds offered the NC diet, but not different to birds on the PC2 or NC+ diets. The BWG of birds on the PC1 diet was greater than that of birds in any other group during the finisher and overall phase (*p* < 0.001). During the starter/grower phase, NC+ resulted in similar BWG to both birds offered PC1 and PC2, whereas the BWG of NC birds was 14%, 10% and 11% lower than birds offered PC1, PC2 and NC+, respectively. There was no significant effect on feed conversion ratio during the starter/grower, finisher and overall phase, but birds offered the PC1 diet had greater BW (*p* < 0.001) at 28 d and 42 d and required significantly fewer (*p* < 0.001) days to reach the target weight of 2.2 kg than birds offered the other three diets. Although not significantly different, phytase superdosing in NC+ diet reduced the days to reach target weight when compared to birds offered the NC. While birds in the PC2 and NC+ groups displayed a similar BW at 28 d (1843 ±7.1 g) and 42 d (3113 ±1.4 g) in comparison, the BW of NC birds at these stages was significantly (*p* < 0.05) reduced by 10% and 5%, respectively. 

DXA results are presented in Table 3. BMD, BMC, bone mass, lean mass and fat mass were significantly greater in birds offered PC1 than birds in any other treatment group. The phytase superdose increased BMC, fat mass and lean mass of birds to similar levels of PC2 birds. Bone mass was increased (*p* = 0.007) by 3% and 7% in birds which received the NC+ treatment compared to those in the PC2 and NC groups. 

### 3.2. Bone Strength

There was no significant difference in the tibia bone strength parameters (weight, diameter, max load, BMD and BMC) measured in birds offered the different experimental diets (Table 4). Although, tibia length was reduced in PC1 birds. 

### 3.3. Digestibility Results

The effect of experimental treatment on the apparent total tract digestibility (ATTD) of various nutrients and minerals in broilers is reported in Table 5. Dry matter digestibility was on average 2.4% greater (*p* = 0.002) in birds offered NC+ diet than birds in all other treatment groups. The digestibility of NDF of birds offered PC1 and PC2 diets was significantly lower (*p* < 0.001) compared to that of birds offered NC+. Gross energy digestibility was highest in NC+ birds and significantly greater (*p* = 0.004) than birds offered PC2 and NC diets. Birds offered NC+ diets displayed higher Ca digestibility than birds in all other treatment groups (*p* < 0.001). The Ca digestibility of birds offered PC1 and NC was similar but both were significantly greater than those offered PC2. The digestibility of K and Mg was greatest in birds offered NC+ (*p* = 0.014, *p* < 0.001) with no difference between other treatment groups (K = 41.6 ± 0.38%, Mg = 33.7 ± 0.35%, respectively). No significant differences in the digestibility of ash, Oil B, N or P were observed between birds offered different diet treatments.

The effects on Apparent ileal digestibility (AID) are presented in Table 5. No significant difference was reported in the AID of DM or ash of birds on the four different treatments.

### 3.4. Jejunum Microbiota

**Figure 1:**
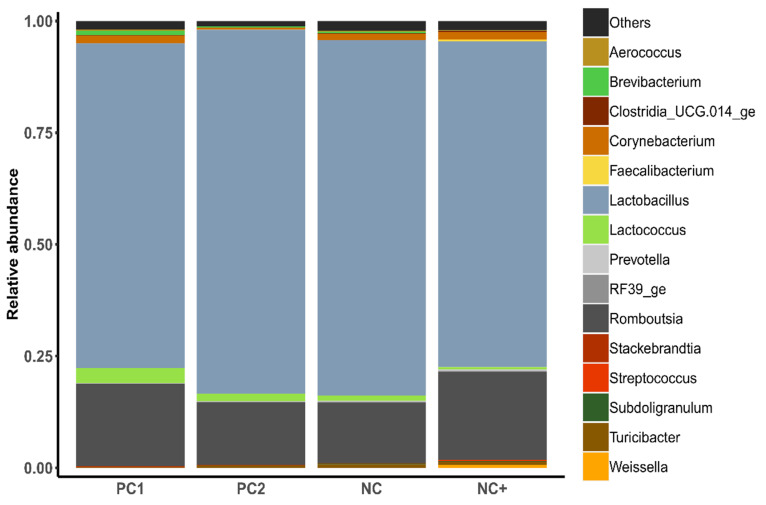
The relative abundance of various microbes in the jejunum of broilers offered the different experimental diets.

The microbial composition of the jejunal samples showed that microbes of the genera *Lactobacillus* and *Romboutsia* had the highest relative abundance in all treatment groups (Figure 1). The effects of the experimental treatments on the relative abundance of genera in the small intestine of broilers are reported in Table 6 and Supplementary Table S1. *Weissella* was significantly (*p* < 0.001) more abundant in birds receiving NC+ compared to all other treatments. Moreover, the NC+ diet resulted in a lower abundance of *Stackebrandtia* compared to PC2 and lower abundance of *Brevibacterium* compared to PC2 and NC diets. Birds offered the PC1 diet showed considerable differences in the microbial composition compared to other treatment groups. Specifically, *Turicibacter* and *Clostridium sensu stricto* 1, (both belonging to *Firmicutes*) were less abundant in birds on the PC1 diet. *Alloprevotella* was less abundant in chickens on the PC1 treatment compared to PC2 and NC.

### 3.5. Nitrogen Balance 

The results of broiler N intake, retention and excretion are presented in Table 7. The N intake and N retention of birds during days 0–28 were significantly lower (*p* < 0.001) for those offered the NC compared to those on any other treatment. Birds offered the NC+ diet displayed similar levels of N intake and retention to birds offered PC1 and PC2. N excretion per day or per kg BW gain during the starter/grower phase did not significantly differ between groups. During the finisher phase (29–42 d), birds offered the PC1 diet displayed significantly higher levels of N retention (17%) compared to birds offered the other three diets. Birds offered the PC1 diet also displayed significantly reduced N excretion per kg BW gain (by 7.04 g/kg) in comparison with those offered the PC2 diet, neither of which were significantly different from N excretion levels of the NC- or NC+-offered birds. N intake and N excretion (g/day) were unaffected by diet treatment during the finisher phase. When considered across the entire study phase (0–42 d), broilers offered the NC diet had reduced N intake compared to PC1 and PC2 birds but similar N intake to birds on the NC+ treatment. The overall N retention per day was greatest in PC1-offered birds compared to those offered any other diet (by 12%). Thus, the N excreted per day (0–42 d) was significantly lower in birds offered the NC diet compared to birds offered the PC2 diet, although neither of which were different to the PC1 and NC+-offered birds. The N excreted per kg BW gain was significantly reduced in PC1-offered birds compared to those offered the PC2 diet. The predicted total N excretion to reach target weight did not significantly differ between groups.

### 3.6. Phosphorus Excretion

The results of broiler P excretion are reported in Table 7. Broilers offered the NC diet displayed the highest P excretion per kg BW gain which was significantly reduced by 1.43 g/kg through the inclusion of a phytase superdose in the starter/grower phase. Although not statistically significant, this trend was observed in the finisher and overall phases. The predicted total P excretion to reach the 2.2 kg target weight did not statistically differ between birds offered the different diet treatments; however, offering the NC diet resulted in the highest total P excretion (13.4 g). 

### 3.7. Phytate, Lower Phytate Esters and Inositol

A significant effect of diet was observed upon the ileal concentration of phytate (IP6), lower phytate esters IP5-IP2 and inositol (Table 5). Ileal concentrations of IP6 were reduced in birds offered NC+, while birds offered PC1 and NC had the highest ileal concentrations of IP6. Again, IP5 concentration was reduced in birds offered NC+, with concentrations higher and similar in birds offered PC2 and NC, with those offered PC1 having the highest IP5 ileal concentrations. Ileal concentrations of IP4 and IP3 were reduced in birds offered the NC in comparison to birds in all other treatments (*p* = 0.009 and *p* = 0.01, respectively). IP4 and IP3 concentrations in the ileal digesta of birds offered PC, PC2 and NC+ were not significantly different (mean = 5188 ± 297 nmol/g and 1050 ± 158 nmol/g, respectively). The concentration of inositol was greater (*p* < 0.001) in the ileal digesta of birds offered the NC+ diet compared to that measured in birds on all other treatments.

## 4. Discussion

### 4.1. Bird Growth Performance

In the current study, growth performance (BW, FI, BWG and days to reach target weight) of birds fed the positive control, traditional wheat/soya-based diet with 500 FTU/kg phytase exceeded that of birds fed the other diet treatments for each phase of study. In fact, the PC1 birds were far ahead of breeder standards, which aim to achieve 3.1 kg BW and 1.5 g/g FCR by 42 d. Although, importantly, birds offered PC2 and NC+ diets, were also to able to meet breeder standards which supports the earlier work which reported that RSM and DDGS diets were successful in maintaining bird performance [29] and suggests these diets may be suitable alternatives to current soya diets. Although, the time to reach the target weight of 2.2 kg was on average 3 days less for birds offered the soya-based diet, which could be of great economic importance to producers when considered over multiple crop cycles and over a number of years. The increased performance observed in the PC1 birds appears to be a result of increased FI, as the digestibility of any nutrients was not significantly greater in these birds. This increased FI observed may be attributed to higher nPP content in the diet. As Akter et al. [43] reported FI corresponded to levels of nPP in the diets provided to broilers and best intakes were observed at higher levels of nPP (4 g/kg) which were actually lower than the formulated levels in the PC1 diet of this study. As expected, birds offered the reduced nutrient specification NC diet, displayed reduced growth performance during the starter/grower, finisher and overall study period in terms of BW, FI and BWG. We suggest this is a result of the reduced nPP (by 20% and 19% in the starter/grower and finisher diets, respectively in comparison to the PC1 diet) and inability to access the phytate-P and other bound nutrients. Previous studies have described similar results that diets reduced in nPP hindered the growth performance of broilers [9,43,44,45]. However, during the starter/grower phase, the FI of birds offered the in the NC diet was reduced compared to NC+ birds, despite a slight increase in nPP (2.8 g/kg vs. 2.6 g/kg). The increased FI of NC+ birds (starter/grower, 7.6%; finisher, 4.0%; and overall, 5.8%) may be as a result of high levels of phytase [23,24,46]. The benefits of phytase addition to the growth performance of broilers have been widely documented with further benefits via the inclusion in superdose levels; for example, Pieniazek et al. [47] reported that higher levels of phytase (2000 FTU/kg) resulted in a 9.1% increase in feed intake and 142 g increase in bird weight compared to birds offered conventional levels of phytase (500 FTU/kg) in corn/soya-based diets. Other work compared the performance of birds offered diets varying in levels of P and phytase (0 and 1500 FTU) and reported that phytase superdosing increased the FI (3.9 vs. 4.1 kg) and body weight gain (2.1 vs. 2.3 kg) of birds offered low-P diets and improved the FCR of all birds [24]. While this current trial does not report differences in FCR, we did observe that phytase superdosing improved the FI and body weight gain of birds by 159 g/day and 192 g/day, respectively, during the starter/grower period and again by 79 g/day and 166 g/day, respectively, when considered from 0 to 42 days, to levels equivalent to or sometimes exceeding birds offered the PC2 diet. This adds to the already widely documented evidence that phytase superdosing in diets reduced in available P improves bird performance [2,9,12,13,23,24,48]. 

### 4.2. Phytase, Phytate Degradation and Nutrient Digestibility

Phytase has been routinely added to broiler diets for over 20 years as poultry lack sufficient levels of this enzyme to break down phytate-P to relieve its antinutritive qualities. The inositol profiles reported in the current work show the concentrations of the inositol phosphates decrease from the higher phosphor-ester forms (IP6) to the lower forms (IP2), which coincides with previous work [49,50,51] and suggests that phytase targets the higher-molecular-weight esters first. The reduced concentrations of IP6 and IP5 in the ileum of birds offered the NC+ diet provides evidence that the increased level of phytase was sufficient to dephosphorylate greater amounts of phytate than diets containing commercial doses or no additional exogenous phytase, as in previous studies [51,52,53]. However, increased levels of IP4 and IP3 in the ileum, as reported here in PC1, PC2 and NC+ birds, have been noted elsewhere [51] and may be due excess Ca in the diet [54], increased pH of ileal digesta [55] or the rate at which different-sized particles travel through the gut at unequal transition rates, i.e., the smaller particles move through faster and are not retained for sufficient digestion [56]. Decreased concentrations of IP4 and IP3 as a result of phytase addition have been reported to occur in the crop and gizzard [53].

The addition of phytase in superdose levels did increase the inositol concentrations in the ileal digesta, which corresponds with the results of other work [51,57,58,59] and suggests that the phytase dose of 1500 FTU/kg was sufficient to degrade dietary phytate to down to lower IP esters and pure inositol. This, along with the increased FI of NC+ birds, may help to explain the improvements to performance observed in these birds in comparison to their NC counterparts. Despite this, the current study only reported small, numeric improvements to P digestibility through phytase superdosing contrary to other work [52,60,61]. This lack of significant difference in P digestibility and higher than expected P digestibility in birds offered the NC diet may be attributed to the levels of endogenous phytase present in the diet. This is supported by the fact the ileal concentrations of the sum of IP, IP6, IP5 and inositol of NC birds were similar to PC1 and PC2 birds, suggesting some degradation of phytate did occur in the NC birds, which increased access to P. While Poulsen et al. [62] also reported high levels of endogenous phytase in the non-heat-treated diets (650 FTU/kg) of pigs, P digestibility was still increased through the additional use of microbial phytase (750 FTU/kg, total phytase activity 1400 FTU/kg). The phytase superdose did appear to alleviate some of the antinutritive effects of phytate and improve the digestibility of DM (1.8%), NDF (3.3%), GE (1.6%), Ca (by 5% compared to PC1 and PC2 and 6.6% compared to NC), K (2.2%) and Mg (4.8%), consistent with previous work [1,2,4,5,6,7,8].

### 4.3. Phytase and N and P Excretion

Contrary to previous work [63] and what may be expecteda reduction in P content of the diet (NC) resulted in greater P excretion than birds offered diets with a higher P content, despite reduced feed intake. This increase in P excretion may be a result of the slight reduction in P digestibility of NC birds. As expected, and in line with the available literature [17,18,64], P excretion was reduced via the inclusion of a phytase superdose. Our study reported a 20% reduction during 0–28 d on a g/kg BW gain basis and a reduction of 15% in total P excretion to 2.2 kg target weight and coincides with the slight improvement observed in P digestibility of NC+ birds. Although the levels of P excretion reported in this study are slightly higher than previously reported, and these studies have achieved greater reductions in P excretion (e.g., 30%) [17,18] using lower doses of phytase (500 FTU/kg), the current work does highlight the benefits of phytase as a feed additive to reduce P losses, improve P utilisation and ultimately reduce the environmental impacts of broiler production. 

Improvements in nutrient retention have been commonly reported as a result of phytase addition in high doses ranging from 1000 to 20,000 FTU/kg [65,66,67,68]. Specifically, an earlier study observed that broilers (aged 21 d) fed with wheat-based diets including phytase displayed increased N retention by 11%, compared with those fed a diet without phytase, due to increased destruction of the phytate molecule [69]. However, in the current study, N retention was not improved by phytase superdosing but corresponded to N intake, in that higher N intake of NC+ birds resulted in greater N retention compared to NC birds. As a result, phytase inclusion also had no effect on N excretion. Though N excretion can be reduced by reducing CP content in the diet [70]. The present work shows that by reducing the CP content in the RSM/DDGS diets, reductions in the total N excretion of birds over their growth period by 15% and 13% for NC and NC+ birds, respectively, were achieved. 

### 4.4. Bone Strength and Body Composition

Improvements to bone strength parameters including increased tibia ash, increased BMD and BMC and increased breaking strength as a result of the inclusion of phytase, have been commonly reported in the available literature [12,22,23,60]. However, the current study found no differences in the tibia bone strength parameters measured. This is despite the observed increased digestibility of Ca and numeric improvements in ATTD of ash achieved through increased phytase content. This suggests that our Ca levels in reduced specification diets (NC and NC+; starter/grower, 7.2; and finisher, 6.1) were sufficient to maintain tibia bone health. This is further supported by the fact that the tibia BMD recorded in this study was similar to, if not slightly greater than, those reported in birds of similar age [12,71,72]. These previous experiments also reported that tibia BMD was reduced in birds fed negative control diets, which is not the case in this study. However, when considered across the entire body, DXA analysis showed improvements to the BMD, BMC, bone mass, lean mass and fat mass by phytase superdosing the NC diet to levels equivalent to PC2 birds, which is in line with previous research [9,12,23,48,73]. Although, as these parameters were still greatest in PC1 birds, this suggests these differences may be attributed to the increased FI and in turn greater intake of nutrients, which is further improved by the addition of phytase superdosing. The differences highlighted by considering the whole-body DXA analysis also suggest that reliance on a sample of an individual bone per subject animal may not be truly reflective of the effects of a treatment upon the composition of an animal. 

### 4.5. Jejunum Microbiota Community

The gut microbiota of chickens is composed of hundreds of microbial species, of which the most common belong to the *Firmicutes*, *Bacteroidetes*, *Proteobacteria* and *Actinobacteria* phyla [27,74], although the structure of the community differs along the gastro-intestinal tract. The present study found the most abundant genera belong to the phyla *Firmicutes* and *Actinobacteria* (Supplementary Table S1). Evidence suggests that the microbial community in the gut can be influenced by dietary ingredients; a recent analysis comparing soyabean and soy-free chicken diets revealed considerable differences between birds with several genera being different across production stages. Specifically, at 7 weeks of age, birds offered soyabean diets had increased abundance of *Firmicutes*, whereas the abundance of *Proteobacteria*, *Actinobacteria* and *Bacteroidetes* was reduced compared to soya-free-fed birds. However, by 12 weeks, this effect was reversed [75]. Contrary to these results, the current study found that the use of RSM/DDGS over the soya-meal-based diet also resulted in increased *Firmicutes*, specifically *Turicibacter,* which has been previously linked to favourable feed conversion in broilers [75] and *Clostridium sensu stricto* 1 compared to birds fed the soya-based diet; both of these bacteria are commonly found in the gut of broilers [73].

There is recent evidence to suggest that phytase superdosing increases the abundance of beneficial bacteria, e.g., *Lactobacillus,* and reduces the abundance of the harmful bacteria in the jejunum (*Pelomonas*, *Helicobacter* and *Pseudomonas)* [27,28], thus potentially positively influencing the microbiota in the gut. The phytase superdose treatment also promoted a shift in the microbial composition of the jejunum digesta, specifically increasing the abundance of *Weissella* spp. (to which probiotic properties are attributed [76]). For birds offered the NC+ treatment, this aligns with the observed improvements in ATTD of fibre and dry matter. As well as this, the abundance of *Stackebrandtia* and *Brevibacterium* (both *Actinobacteria*) were markedly reduced compared to the levels found in birds offered the other treatments. This could prove beneficial as *Actinobacteria* have been identified as important and common in the digestive tract of other high-producing broilers [77].

While not considered in the present study, there is evidence available suggesting that dietary ingredients can influence the gut morphology of broilers. For example, Qaisrani et al. [78] reported birds fed soyabean meal diets had increased villi height (VH) and increased villus height to crypt depth ratio (VH:CD) compared with those fed RSM and maise glutendiets, which may be an additional reason for the improved performance of birds offered the PC1 treatment in the current trial. Similar alterations in gut morphology including increased VH and CD; changes in VH:CD; increased goblet cells; and reduced epithelial thickness have been reported as a result of phytase inclusion [79]. The work of Moita et al. [27] reported that these changes increased with increasing levels of phytase (500–4000 FTU/kg), all of which could provide beneficial effects on the further absorption of nutrients and may be important to consider in further studies.

## 5. Conclusions

Traditional, soya-based diets with commercial doses of exogenous phytase yielded the best growth performance of birds. However, the current study provides evidence to suggest that the inclusion of a phytase superdose to the sustainable RSM/DDGS diet with reduced nutrient content can improve the growth performance of birds to levels comparable to those fed to nutrient recommendations and maintain performance at breeder standards therefore may be suitable alternative sustainable diets. The use of phytase at commercial and superdosing levels shows potential as a useful tool to improve the performance of broilers offered a more sustainable, lower-cost diet and to reduce N and P excretion via enhancing the digestibility of various nutrients or modifying the gut microbiota with the potential to improve gut health and enhance nutrient absorption, ultimately reducing the excess use of inorganic-P in diets, reducing feed costs to producers and reducing the environmental impacts of broiler production. When formulating broiler diets, consideration should be given to the balance of reducing N and P excretion, animal performance, the availability and sustainability of dietary ingredients and the cost of diet production. Consideration should also be given to the activity of endogenous phytase and further studies could potentially investigate how this may interact with the benefits of additional exogenous phytase. This information could be vital for producers who produce their own non-heat-treated diets. 

## Figures and Tables

**Table 1:** Formulated composition of experimental treatments.

	Starter/Grower (0–28 d)				Finisher (29–42 d)			
	PC1	PC2	NC	NC+	PC1	PC2	NC	NC+
Wheat, g/kg	602	563	604	604	608	476	518	517
Maize DDGS, g/kg	—	35	35	35	—	100	100	100
Soyabean meal, g/kg	305	280	260	260	279	197	176	176
Rapeseed meal extract, g/kg	—	25	25	25	—	100	100	100
Soya oil, g/kg	24	27.9	11.4	11.5	52.7	68.8	52.2	52.3
Salt, g/kg	3.3	3	1.9	1.9	2.5	1.8	1.4	1.4
Limestone, g/kg	10.4	10.8	10.6	10.6	9.4	10.7	10.5	10.5
Mono-Dicalcium Phosphate, g/kg	11.1	10.1	7	7	8.2	5	1.9	1.9
Sodium Bicarbonate, g/kg	2	2	3	3	2	2	2	2
Premix ^1^, g/kg	25	25	25	25	25	25	25	25
L-lysine HCl, g/kg	5.8	6.2	6.4	6.4	3.8	5	5.1	5.1
Methionine, g/kg	5	4.9	4.4	4.4	4	3.7	3.1	3.1
Threonine, g/kg	3.3	3.3	3	3	2.1	2.1	1.8	1.8
Quantum Blue 5G (1500 FTU/kg)	—	—	—	0.3	—	—	—	0.3
Quantum Blue 5G (500 FTU/kg)	0.1	0.1	—	—	0.1	0.1	—	—
Formulated composition								
Crude protein, g/kg	215	215	209	209 (215)	200	200	194	194 (200)
Metabolizable energy, MJ/kg	12.6	12.6	12.2	12.2 (12.6)	13.4	13.4	13.1	13.1 (13.4)
Dry matter, g/kg	857	858	856	856	859	866	863	863
Calcium, g/kg	9.6	9.6	7.2	7.2 (9.6)	8.5	8.5	6.1	6.1 (8.5)
Phosphorus (P), g/kg	7.2	7.3	5.1	5.1 (7.3)	6.4	6.7	4.5	4.5 (6.7)
Phytate- P, g/kg	2.4	2.5	2.5	2.5	2.3	2.6	2.6	2.6
Non Phytate-P (nPP), g/kg	4.8	4.8	2.6	2.6 (4.8)	4.1	4.1	1.9	1.9 (4.1)
Ca: nPP, g/kg	2	2	2.8	2.8 (2.0)	2.1	2.1	3.2	3.2 (2.1)
Lysine, g/kg	14.3	14.3	14	14	12	12.1	11.8	11.8
Methionine, g/kg	7.6	7.6	7	7	6.5	6.4	5.8	5.8
Threonine, g/kg	10.1	10.2	9.6	9.6	8.5	8.7	8.1	8.1
Tryptophan, g/kg	2.4	2.4	2.1	2.1	2.1	2.1	1.8	1.8

^1^ Vitamin/mineral premix supplied per kilogram of diet: The trace mineral/vitamin mixture supplied (per kg feed): retinol 3.6 mg, cholecalciferol 0.125 mg, α-tocopherol 50 mg, thiamine 2 mg, riboflavin 7 mg, vitamin K 3 mg, pyridoxine 5 mg, nicotinic acid 50 mg, calcium pantothenate 15 mg, folic acid 1 mg, biotin 0.2 mg, cobalamin 15 mg, manganese 100 mg, iron 80 mg, zinc 80 mg, copper 10 mg, iodine 1 mg, cobalt 0.5 mg, selenium 0.2 mg, and molybdenum 0.5 mg. Figures presented within “()” indicate the levels of nutrients available with Quantum Blue 5G inclusion.

**Table 2:** Analysed nutrient composition of experimental starter/grower and finisher diets.

	Starter/Grower				Finisher			
	PC1	PC2	NC	NC+	PC1	PC2	NC	NC+
Gross energy, MJ/kg	18.89	19.07	18.89	18.56	19.40	20.16	19.95	19.93
Apparent metabolizable energy, MJ/kg	12.67	12.79	12.5	12.61	13.33	13.84	13.52	13.86
Dry matter, g/kg	862	860	863	854	877	884	878	882
Ether extract *, g/kg	48.1	55.7	47.0	38.9	75.1	105.0	90.2	92.0
Ash *, g/kg	69.0	68.1	63.6	63.5	60.9	62.4	60.9	57.2
Neutral detergent fibre *, g/kg	159	169.7	181.6	163.8	125.2	192.0	203.8	179.0
Crude protein *, g/kg	209	220	208	214	199	205	201	201
Calcium, g/kg	9.8	9.4	8.4	9.3	9.7	8.6	8.8	7.5
Phosphorus, g/kg	6.0	5.8	5.4	5.1	5.0	5.3	4.9	4.9
Phytate-P, g/kg	2.5	2.5	2.6	2.5	2.3	2.5	2.7	2.6
Non phytate- phosphorus (nPP), g/kg	3.5	3.3	2.8	2.6	2.7	2.8	2.2	2.3
Lysine, g/kg	15.1	15.1	13.7	14.8	12.8	12.5	11.5	11.5
Methionine, g/kg	7.0	7.0	6.2	6.8	6.4	6.6	5.9	6.1
Threonine, g/kg	10.6	10.6	9.5	10.1	9.1	9.2	8.9	9.1
Tryptophan, g/kg	2.8	2.8	2.4	2.6	2.4	2.3	2.3	2.3
Quantum Blue phytase, FTU/kg	541	563	-	1390	522	544	-	1170
Endogenous phytase, FTU/kg	206	407	550	290	344	332	365	640
Total phytase activity, FTU/kg	747	970	550	1680	866	876	365	1810

* Dry matter basis.

**Table 3:** Broiler production performance and body composition of birds offered the different dietary treatments during the starter/grower, finisher and overall phases.

	PC1	PC2	NC	NC+	Sem	*p*
Weight day 28, g	1910 ^c^	1838 ^b^	1656 ^a^	1848 ^b^	22.7	<0.001
Weight day 42, g	3447 ^c^	3112 ^b^	2948 ^a^	3114 ^b^	53.8	<0.001
Days to reach target weight (2.2 kg)	27.2 ^a^	30.1 ^b^	31.9 ^b^	30.2 ^b^	0.74	<0.001
Starter/Grower (days 0–28)						
FI, g	2400 ^c^	2258 ^b^	2089 ^a^	2248 ^b^	43.53	<0.001
BWG, g	1870 ^c^	1798 ^b^	1616 ^a^	1808 ^bc^	22.74	<0.001
FCR, g/g	1.28	1.26	1.29	1.24	0.020	0.297
Finisher (days 28–42)						
FI, g	2232 ^b^	2045 ^ab^	1983 ^a^	2062 ^ab^	59.1	0.036
BWG, g	1537 ^b^	1274 ^a^	1292 ^a^	1266 ^a^	42.2	<0.001
FCR, g/g	1.46	1.61	1.54	1.64	0.052	0.076
Overall (days 0–42)						
FI, g	4633 ^c^	4327 ^b^	4072 ^a^	4309 ^ab^	79.7	<0.001
BWG, g	3407 ^c^	3071 ^b^	2908 ^a^	3074 ^b^	53.8	<0.001
FCR, g/g	1.36	1.41	1.40	1.40	0.019	0.277
Whole-body DXA						
BMD, g/cm^2^	0.43 ^b^	0.40 ^a^	0.39 ^a^	0.39 ^a^	0.004	0.003
BMC, g	93.16 ^c^	80.48 ^b^	75.77 ^a^	81.14 ^b^	1.05	<0.001
Bone mass, g	219 ^d^	204 ^b^	197 ^a^	211 ^c^	2.0	0.007
Lean mass, g	2136 ^c^	1879 ^b^	1791 ^a^	1849 ^ab^	24.97	<0.001
Fat mass (g)	1068 ^c^	943 ^a^	935 ^a^	994 ^b^	13.8	0.003

^a,b,c^ in the same row indicate significant difference *p* < 0.05; Days to reach target weight (2.2 kg) = 2.2 kg/BWG, g (0–42 d)/42; Abbreviations: FI, feed intake; BWG, body weight gain; FCR, feed conversion ratio; BMD, bone mineral density; BMC, bone mineral content.

**Table 4:** Tibia bone strength parameters of broilers offered the different experimental diets.

Tibia Measurements	PC1	PC2	NC	NC+	SEM	*p*
Weight, g	25.3	23.7	24.0	24.6	0.74	0.544
Diameter, mm	8.83	8.60	8.88	8.80	0.204	0.710
Length, mm	110.0 ^a^	113.5 ^b^	113.7 ^b^	112.2 ^b^	0.879	0.046
Max load, kg	42.5	40.5	41.3	40.3	2.38	0.930
Bone mineral density, g/cm^2^ *	0.33	0.35	0.33	0.37	0.009	0.498
Bone mineral content, g *	0.60	0.67	0.62	0.70	0.02	0.369

^a ,b^ in the same row indicate significant difference *p* < 0.05 * As measured by DXA.

**Table 5:** The apparent total tract digestibility and apparent ileal digestibility of various nutrients and ileal IP ester (IP6-IP2) and inositol concentrations of broilers offered different experimental diets.

Apparent Total Tract Digestibility (ATTD), %	PC1	PC2	NC	NC+	SEM	*p*
Dry matter	74.9 ^a^	74.6 ^a^	74.2 ^a^	76.4 ^b^	0.40	0.002
Gross energy	78.0 ^ab^	77.8 ^a^	77.0 ^a^	79.0 ^b^	0.35	0.004
Ash	45.9	44.9	45.6	47.3	0.67	0.088
Oil B	82.7	83.5	80.3	81.6	0.86	0.056
Neutral detergent fibre	55.5 ^a^	57.4 ^ab^	58.5 ^bc^	59.7 ^c^	0.67	<0.001
Calcium	50.4 ^b^	47.6 ^a^	48.2 ^ab^	54.2 ^c^	0.88	<0.001
Potassium	41.0 ^a^	40.9 ^a^	41.6 ^a^	43.4 ^b^	0.58	0.014
Magnesium	33.2 ^a^	33.9 ^a^	33.6 ^a^	38.4 ^b^	0.53	<0.001
Nitrogen	70.2	70.1	69.6	70.8	0.79	0.786
Phosphorus	76.2	76.5	75.7	78.2	0.01	0.400
Apparent ileal digestibility (AID), %						
Dry matter	72.9	68.2	70.3	70.8	1.01	0.757
Ash	48.8	47.4	48.0	49.6	1.71	0.973
Ileal concentrations, nmol/g						
IP6	36,275 ^c^	25,674 ^b^	39,895 ^c^	8539 ^a^	2803.7	<0.001
IP5	11,383 ^c^	8033 ^b^	6969 ^b^	3718 ^a^	622.5	<0.001
IP4	5530 ^b^	5033 ^b^	2450 ^a^	5000 ^b^	366.6	0.009
IP3	920 ^b^	1005 ^b^	220 ^a^	1226 ^b^	122.7	0.01
IP2	460 ^c^	377 ^c^	138 ^a^	251 ^b^	35.8	0.009
IP sum *	54,453 ^b^	40,067 ^b^	49,602 ^b^	18,639 ^a^	5280	<0.001
Inositol	1083 ^a^	2594 ^a^	3434 ^a^	6504 ^b^	532.2	<0.001

^a,b,c^ in the same row indicate significant difference *p* < 0.05; * Summation of inositol phosphates 2–6.

**Table 6:** Differences in the relative abundance of genera in the jejunal digesta of broilers offered different experimental diets.

Relative Abundance of Genera (Phylum), %	PC1	PC2	NC	NC+	SEM	*p*
*Weissella* (*Firmicutes*)	0.006 ^a^	0.005 ^a^	0.004 ^a^	0.398 ^b^	0.041	<0.001
*Turicibacter* (*Firmicutes*)	0.064 ^a^	0.421 ^b^	0.379 ^b^	0.471 ^b^	0.060	0.002
*Clostridium sensu stricto* 1 (*Firmicutes*)	0.006 ^a^	0.038 ^b^	0.057 ^b^	0.053 ^b^	0.007	0.002
*Stackebrandtia* (*Actinobacteriota*)	0.167 ^ab^	0.055 ^b^	0.041 ^ab^	0.014 ^a^	0.021	0.030
*Alloprevotella* (*Bacteroidota*)	0.001 ^a^	0.017 ^b^	0.027 ^b^	0.046 ^ab^	0.006	0.038
*Brevibacterium* (*Actinobacteriota*)	0.667 ^ab^	0.226 ^b^	0.227 ^b^	0.061 ^a^	0.092	0.041

^a,b^ in the same row indicate significant difference *p* < 0.05.

**Table 7:** Results of N balance and P excretion of broilers fed experimental diets (PC1, PC2, NC and NC+) during the starter/grower (0–28 d), finisher (29–42 d) and overall (0–42 d) phases.

	PC1	PC2	NC	NC+	SEM	*p*
**N balance**						
Starter/Grower (0–28 d)						
N intake (g/day)	2.86 ^b^	2.84 ^b^	2.48 ^a^	2.74 ^b^	0.074	<0.001
N retention (g/day)	1.92 ^b^	1.85 ^b^	1.66 ^a^	1.86 ^b^	0.033	<0.001
N excretion (g/day)	0.94	0.99	0.82	0.88	0.062	0.052
N excretion (g/kg gain)	14.07	15.5	14.17	13.67	0.956	0.266
Finisher (29–42 d)						
N intake (g/day)	5.08	4.85	4.56	4.76	0.198	0.090
N retention (g/day)	3.16 ^b^	2.62 ^a^	2.66 ^a^	2.60 ^a^	0.123	<0.001
N excretion (g/day)	1.92	2.23	1.90	2.16	0.176	0.169
N excretion (g/kg gain)	17.57 ^a^	24.61 ^b^	20.72 ^ab^	24.25 ^ab^	2.294	0.015
Overall (0–42 d)						
N intake (g/day)	3.60 ^b^	3.49 ^b^	3.17 ^a^	3.41 ^ab^	0.088	<0.001
N retention (g/day)	2.34 ^b^	2.11 ^a^	1.99 ^a^	2.11 ^a^	0.052	<0.001
N excretion (g/day)	1.26 ^ab^	1.38 ^b^	1.18 ^a^	1.30 ^ab^	0.065	0.032
N excretion (g/kg gain)	15.58 ^a^	18.89 ^b^	17.02 ^ab^	17.81 ^ab^	0.918	0.010
N excretion to reach target weight of 2.2 kg (g) *	25.52	29.93	25.99	26.48	1.774	0.07
P excretion (g/day)	0.44	0.43	0.42	0.38	0.029	0.208
P excretion 0–28 d (g/kg gain)	6.57 ^ab^	6.67 ^ab^	7.29 ^b^	5.86 ^a^	0.46	0.033
P excretion 29–42 d (g/kg gain)	4.00	4.70	4.56	4.19	0.31	0.097
P excretion 0–42 d (g/kg gain)	5.41	5.86	6.08	5.17	0.393	0.109
Total P excretion to reach target weight of 2.2 kg (g) *	11.9	12.9	13.4	11.4	0.865	0.109

^a,b^ in the same row indicate significant difference *p* < 0.05; * Based on N excretion per day for 0–28 d phase and number of days required to reach 2.2 kg.

## Data Availability

Data are available in a publicly accessible repository.

## Supplementary Materials

The following supporting information can be downloaded at: https://www.mdpi.com/article/10.3390/ani12131669/s1, Table S1: Mean relative abundance of microbial genera in the jejunal digestive tract of broilers offered different experimental diets.

## References

[1] Ravindran V., Cowieson A.J., Selle P.H. (2008). Influence of dietary electrolyte balance and microbial phytase on growth performance, nutrient utilization, and excreta quality of broiler chickens. Poult. Sci..

[2] Walk C.L., Rao S.R. (2020). Dietary phytate has a greater anti-nutrient effect on feed conversion ratio compared to body weight gain and greater doses of phytase are required to alleviate this effect as evidenced by prediction equations on growth performance, bone ash and phytate degradation in broilers. Poult. Sci..

[3] Abbasi F., Fakhur-un-Nisa T., Liu J., Luo X., Abbasi I.H.R. (2019). Low digestibility of phytate phosphorus, their impacts on the environment, and phytase opportunity in the poultry industry. Environ. Sci. Pollut. Res..

[4] Selle P.H., Ravindran V., Caldwell A., Bryden W.L. (2000). Phytate and phytase: Consequences for protein utilisation. Nutr. Res. Rev..

[5] Santos F.R., Hruby M., Pierson E.E.M., Remus J.C., Sakomura N.K. (2008). Effect of phytase supplementation in diets on nutrient digestibility and performance in broiler chicks. J. Appl. Poult. Res..

[6] Attia Y.A., Bovera F., Al-Harthi M.A., El-Din A.E.R.E., Said Selim W. (2021). Supplementation of microbial and fungal phytases to low protein and energy diets: Effects on productive performance, nutrient digestibility, and blood profiles of broilers. Agriculture.

[7] Dersjant-Li Y., Dusel G. (2019). Increasing the dosing of a Buttiauxella phytase improves phytate degradation, mineral, energy, and amino acid digestibility in weaned pigs fed a complex diet based on wheat, corn, soybean meal, barley, and rapeseed meal. J. Anim. Sci..

[8] Dersjant-Li Y., Davin R., Christensen T., Kwakernaak C. (2021). Effect of two phytases at two doses on performance and phytate degradation in broilers during 1–21 days of age. PLoS ONE.

[9] Walters H.G., Coelho M., Coufal C.D., Lee J.T. (2019). Effects of increasing phytase inclusion levels on broiler performance, nutrient digestibility, and bone mineralization in low-phosphorus diets. J. Appl. Poult. Res..

[10] Powell S., Bidner T.D., Southern L.L. (2011). Phytase supplementation improved growth performance and bone characteristics in broilers fed varying levels of dietary calcium. Poult. Sci..

[11] Walk C.L., Bedford M.R., Santos T.S., Paiva D., Bradley J.R., Wladecki H., Honaker C., McElroy A.P. (2013). Extra-phosphoric effects of superdoses of a novel microbial phytase. Poult. Sci..

[12] Leyva-Jimenez H., Alsadwi A.M., Gardner K., Voltura E., Bailey C.A. (2019). Evaluation of high dietary phytase supplementation on performance, bone mineralization, and apparent ileal digestible energy of growing broilers. Poult. Sci..

[13] Al-Qahtani M., Ahiwe E.U., Abdallh M.E., Chang’a E.P., Gausi H., Bedford M.R., Iji P.A. (2021). Endogenous enzyme activities and tibia bone development of broiler chickens fed wheat-based diets supplemented with xylanase, β-glucanase and phytase. Anim. Biosci..

[14] Sukumar D., Jalaludeen A., Cyriac S. (2013). Effect of mitigating environmental pollution by incorporation of phytase enzyme in feed of (Athulya) layer chicken. Wayamba J. Anim. Sci..

[15] Musapuor A., Afsharmanesh M., Shahrbabak H.M. (2006). Use of microbial phytase for decrease of pollutant due to environmental poultry excreta phosphorus. Int. J. Agric. Biol..

[16] Bougouin A., Appuhamy J.A.D.R.N., Kebreab E., Dijkstra J., Kwakkel R.P., France J. (2014). Effects of phytase supplementation on phosphorus retention in broilers and layers: A meta-analysis. Poult. Sci..

[17] Lalpanmawia H., Elangovan A.V., Sridhar M., Shet D., Ajith S., Pal D.T. (2014). Efficacy of phytase on growth performance, nutrient utilization and bone mineralization in broiler chicken. Anim. Feed Sci. Technol..

[18] Puppala K.R., Buddhiwant P.G., Agawane S.B., Kadam A.S., Mote C.S., Lonkar V.D., Khire J.M., Dharne M.S. (2021). Performance of *Aspergillus niger* (NCIM 563) phytase based feed supplement for broiler growth and phosphorus excretion. Biocatal. Agric. Biotechnol..

[19] Humer E., Schwarz C., Schedle K. (2015). Phytate in pig and poultry nutrition. J. Anim. Physiol. Anim. Nutr..

[20] Scholey D.V., Morgan N.K., Riemensperger A., Hardy R., Burton E.J. (2018). Effect of supplementation of phytase to diets low in inorganic phosphorus on growth performance and mineralization of broilers. Poult. Sci..

[21] Babatunde O.O., Bello A., Dersjant-Li Y., Adeola O. (2021). Evaluation of the responses of broiler chickens to varying concentrations of phytate phosphorus and phytase. Ⅰ. Starter phase (day 1–11 post hatching). Poult. Sci..

[22] Fernandes J.I.M., Horn D., Ronconi E.J., Buzim R., Lima F.K., Pazdiora D.A. (2019). Effects of Phytase Superdosing on Digestibility and Bone Integrity of Broilers. J. Appl. Poult. Res..

[23] Walk C.L., Santos T.T., Bedford M.R. (2014). Influence of superdoses of a novel microbial phytase on growth performance, tibia ash, and gizzard phytate and inositol in young broilers. Poult. Sci..

[24] Lee S.A., Nagalakshmi D., Raju M.V., Rao S.V.R., Bedford M.R. (2017). Effect of phytase superdosing, myo-inositol and available phosphorus concentrations on performance and bone mineralisation in broilers. Anim. Nutr..

[25] Infanzón B., Herrmann K.R., Hofmann I., Willbold S., Ruff A.J., Schwaneberg U. (2022). Phytase blends for enhanced phosphorous mobilization of deoiled seeds. Enzyme Microb. Technol..

[26] Zanu H.K., Keerqin C., Kheravii S.K., Morgan N., Wu S.B., Bedford M.R., Swick R.A. (2020). Influence of meat and bone meal, phytase, and antibiotics on broiler chickens challenged with subclinical necrotic enteritis: 2. intestinal permeability, organ weights, hematology, intestinal morphology, and jejunal gene expression. Poult. Sci..

[27] Moita V.H.C., Duarte M.E., Kim S.W. (2021). Supplemental Effects of Phytase on Modulation of Mucosa-Associated Microbiota in the Jejunum and the Impacts on Nutrient Digestibility, Intestinal Morphology, and Bone Parameters in Broiler Chickens. Animals.

[28] Ptak A., Bedford M.R., Świątkiewicz S., Żyła K., Józefiak D. (2015). Phytase modulates ileal microbiota and enhances growth performance of the broiler chickens. PLoS ONE.

[29] Ball M.E., Beattie V., Hayes H., Lawlor P., Magowan E. The use of enzymes in pigs and broiler diets containing low soya and varying levels of energy and amino acids. Proceedings of the 67th EAAP Annual Meeting.

[30] Hollands C. (1986). The animals (scientific procedures) act 1986. Lancet Lond..

[31] Crenshaw T.D., Peo E.R., Lewis A.J., Moser B.D. (1981). Bone strength as a trait for assessing mineralization in swine: A critical review of techniques involved. J. Anim. Sci..

[32] AOAC (1995). Official Methods of Analysis.

[33] Van Soest P.J. (1963). The use of detergent in the analysis of fibrous feeds. II. A rapid method for the determination of fiber and lignin. J. Assoc. Off. Agric. Chem..

[34] Leone J.L. (1973). Collaborative study of the quantitative determination of titanium dioxide in cheese. AOAC.

[35] Peddie J., Dewar W.A., Gilbert A.B., Waddington D. (1982). The use of titanium dioxide for determining apparent ileal digestibility in mature domestic fowls (*Gallus domesticus*). J. Agric. Sci..

[36] European Commission Commission regulation (EC) (2009). No 152/2009 of 27 January 2009 laying down the methods of sampling and analysis for the official control of feed. Off. J. Eur. Union..

[37] Lu H., Kühn I., Bedford M.R., Whitfield H., Brearley C., Adeola O., Ajuwon K.M. (2019). Effect of phytase on intestinal phytate breakdown, plasma inositol concentrations, and glucose transporter type 4 abundance in muscle membranes of weanling pigs. J. Anim. Sci..

[38] Whitfield H., White G., Sprigg C., Riley A.M., Potter B.V., Hemmings A.M., Brearley C.A. (2020). An ATP-responsive metabolic cassette comprised of inositol tris/tetrakisphosphate kinase 1 (ITPK1) and inositol pentakisphosphate 2-kinase (IPK1) buffers diphosphosphoinositol phosphate levels. Biochem. J..

[39] Madsen C.K., Brearley C.A., Brinch-Pedersen H. (2019). Lab-scale preparation and QC of phytase assay substrate from rice bran. Anal. Biochem..

[40] Schiavone A., Guo K., Tassone S., Gasco L., Hernandez E., Denti R., Zoccarato I. (2008). Effects of a natural extract of chestnut wood on digestibility, performance traits, and nitrogen balance of broiler chicks. Poult. Sci..

[41] Kozich J.J., Westcott S.L., Baxter N.T., Highlander S.K., Schloss P.D. (2013). Development of a dual-index sequencing strategy and curation pipeline for analyzing amplicon sequence data on the MiSeq Illumina sequencing platform. Appl. Environ. Microbiol..

[42] Hugerth L.W., Wefer H.A., Lundin S., Jakobsson H.E., Lindberg M., Rodin S., Engstrand L., Andersson A.F. (2014). DegePrime, a program for degenerate primer design for broad-taxonomic-range PCR in microbial ecology studies. Appl. Environ. Microbiol..

[43] Akter M., Graham H., Iji P.A. (2019). Response of broiler chickens to diets containing different levels of sodium with or without microbial phytase supplementation. J. Anim. Sci. Technol..

[44] Faridi A., Gitoee A., France J. (2015). A meta-analysis of the effects of nonphytate phosphorus on broiler performance and tibia ash concentration. Poult. Sci..

[45] Fan L., He Z.Z., Ao X., Sun W.L., Xiao X., Zeng F.K., Wang Y.C., He J. (2019). Effects of residual superdoses of phytase on growth performance, tibia mineralization, and relative organ weight in ducks fed phosphorus-deficient diets. Poult. Sci..

[46] Lima G.S., Lima M.R., Gomes G.A., Cavalcante D.T., Guerra R.R., da Silva J.H.V., Cardoso A.S., Kaneko I.N., Costa F.G.P. (2021). Superdosing of bacterial phytase (EC 3.1. 3.26) in broiler diets with reduced levels of digestible amino acids. Livest. Sci..

[47] Pieniazek J., Smith K.A., Williams M.P., Manangi M.K., Vazquez-Anon M., Solbak A., Miller M., Lee J.T. (2017). Evaluation of increasing levels of a microbial phytase in phosphorus deficient broiler diets via live broiler performance, tibia bone ash, apparent metabolizable energy, and amino acid digestibility. Poult. Sci..

[48] Manobhavan M., Sridhar M., Ajith S., Shet D., Pal D.T., Gowda N.K.S., Elangovan A.V. (2015). Efficacy of fungal phytase on growth performance and bone mineralization in broiler chicken. Anim. Nutr. Feed Technol..

[49] Wyss M., Brugger R., Kronenberger A., Rémy R., Fimbel R., Oesterhelt G., Lehmann M., Van Loon A.P. (1999). Biochemical characterization of fungal phytases (myo-inositol hexakisphosphate phosphohydrolases): Catalytic properties. Appl. Environ. Microbiol..

[50] Cowieson A.J., Wilcock P., Bedford M.R. (2011). Super-dosing effects of phytase in poultry and other monogastrics. World’s Poult. Sci. J..

[51] Gautier A.E., Walk C.L., Dilger R.N. (2018). Effects of a high level of phytase on broiler performance, bone ash, phosphorus utilization, and phytate dephosphorylation to inositol. Poult. Sci..

[52] Beeson L.A., Walk C.L., Bedford M.R., Olukosi O.A. (2017). Hydrolysis of phytate to its lower esters can influence the growth performance and nutrient utilization of broilers with regular or super doses of phytase. Poult. Sci..

[53] Walk C.L., Bedford M.R., Olukosi O.A. (2018). Effect of phytase on growth performance, phytate degradation and gene expression of myo-inositol transporters in the small intestine, liver and kidney of 21 day old broilers. Poult. Sci..

[54] Bedford M., Rousseau X. (2017). Recent findings regarding calcium and phytase in poultry nutrition. Anim. Prod. Sci..

[55] Pontoppidan K., Glitsoe V., Guggenbuhl P., Quintana A.P., Nunes C.S., Pettersson D., Sandberg A.S. (2012). In vitro and in vivo degradation of myo-inositol hexakisphosphate by a phytase from *Citrobacter braakii*. Arch. Anim. Nutr..

[56] Carré B. (2004). Causes for variation in digestibility of starch among feedstuffs. World’s. Poult. Sci. J..

[57] Zeller E., Schollenberger M., Witzig M., Shastak Y., Kühn I., Hoelzle L.E., Rodehutscord M. (2015). Interactions between supplemented mineral phosphorus and phytase on phytate hydrolysis and inositol phosphates in the small intestine of broilers. Poult. Sci..

[58] Cowieson A.J., Aureli R., Guggenbuhl P., Fru-Nji F. (2014). Possible involvement of myo-inositol in the physiological response of broilers to high doses of microbial phytase. Anim. Poult. Sci..

[59] Vieira S.L., Anschau D.L., Serafini N.C., Kindlein L., Cowieson A.J., Sorbara J.O.B. (2015). Phosphorus equivalency of a *Citrobracter braakii* phytase in broilers. J. Appl. Poult. Res..

[60] Manobhavan M., Elangovan A.V., Sridhar M., Shet D., Ajith S., Pal D.T., Gowda N.K.S. (2016). Effect of super dosing of phytase on growth performance, ileal digestibility and bone characteristics in broilers fed corn–soya-based diets. J. Anim. Physiol. Anim. Nutr..

[61] Woyengo T.A., Wilson J. (2019). Enhancing nutrient utilization in maize for broiler chickens by superdosing phytase. Anim. Feed Sci. Technol..

[62] Poulsen H.D., Carlson D., Nørgaard J.V., Blaabjerg K. (2010). Phosphorus digestibility is highly influenced by phytase but slightly by calcium in growing pigs. Livest. Sci..

[63] Srikanthithasan K., Macelline S.P., Wickramasuriya S.S., Tharangani H., Jayasena D.D., Heo J.M. (2019). Effects of adding phytase from *aspergillus niger* to a low phosphorus diet on growth performance, tibia characteristics, phosphorus excretion, and meat quality of broilers 35 days after hatching. Poult. Sci. J..

[64] Tsai T.C., Dove R., Bedford M.R., Azain M.J. (2020). Effect of phytase on phosphorous balance in 20-kg barrows fed low or adequate phosphorous diets. Anim. Nutr..

[65] Bento M.H.L., Pedersen C., Plumstead P.W., Salmon L., Nyachoti C.M., Bikker P. (2012). Dose response of a new phytase on dry matter, calcium, and phosphorus digestibility in weaned piglets. J. Anim. Sci..

[66] Adedokun S.A., Owusu-Asiedu A., Ragland D., Plumstead P., Adeola O. (2015). The efficacy of a new 6-phytase obtained from *Buttiauxella* spp. expressed in *Trichoderma reesei* on digestibility of amino acids, energy, and nutrients in pigs fed a diet based on corn, soybean meal, wheat middlings, and corn distillers’ dried grains with solubles. Sci. J. Anim. Sci..

[67] Zeng Z., Li Q., Tian Q., Zhao P., Xu X., Yu S., Piao X. (2015). Super high dosing with a novel *Buttiauxella* phytase continuously improves growth performance, nutrient digestibility, and mineral status of weaned pigs. Biol. Trace Elem. Res..

[68] Dersjant-Li Y., Wealleans A.L., Barnard L.P., Lane S. (2017). Effect of increasing *Buttiauxella* phytase dose on nutrient digestibility and performance in weaned piglets fed corn or wheat based diets. Anim. Feed Sci. Technol..

[69] Afsharmanesh M., Scott T.A., Silversides F.G. (2008). Effect of wheat type grinding, heat treatment and phytase supplementation on growth efficiency and nutrient utilization of wheat-based diets for broilers. Can. J. Anim. Sci..

[70] Attia Y.A., Bovera F., Wang J., Al-Harthi M.A., Kim W.K. (2020). Multiple amino acid supplementations to low-protein diets: Effect on performance, carcass yield, meat quality and nitrogen excretion of finishing broilers under hot climate conditions. Animals.

[71] Angel R., Saylor W.W., Mitchell A.D., Powers W., Applegate T.J. (2006). Effect of dietary phosphorus, phytase, and 25-hydroxycholecalciferol on broiler chicken bone mineralization, litter phosphorus, and processing yields. Poult. Sci..

[72] Chung T.K., Rutherfurd S.M., Thomas D.V., Moughan P.J. (2013). Effect of two microbial phytases on mineral availability and retention and bone mineral density in low-phosphorus diets for broilers. Br. Poult. Sci..

[73] Hakami Z., Al Sulaiman A.R., Alharthi A.S., Casserly R., Bouwhuis M.A., Abudabos A.M. (2022). Growth performance, carcass and meat quality, bone strength, and immune response of broilers fed low-calcium diets supplemented with marine mineral complex and phytase. Poult. Sci..

[74] Diaz Carrasco J.M., Casanova N.A., Fernández Miyakawa M.E. (2019). Microbiota, gut health and chicken productivity: What is the connection?. Microorganisms.

[75] Lourenco J.M., Rothrock Jr M.J., Fluharty F.L., Callaway T.R. (2019). The successional changes in the gut microbiome of pasture-raised chickens fed soy-containing and soy-free diets. Front. Sustain. Food Syst..

[76] Fusco V., Quero G.M., Cho G.S., Kabisch J., Meske D., Neve H., Bockelmann W., Franz C.M. (2015). The genus Weissella: Taxonomy, ecology and biotechnological potential. Front. Microbiol..

[77] Xiao Y., Xiang Y., Zhou W., Chen J., Li K., Yang H. (2017). Microbial community mapping in intestinal tract of broiler chicken. Poult. Sci..

[78] Qaisrani S.N., Van Krimpen M.M., Verstegen M.W.A., Hendriks W.H., Kwakkel R.P. (2020). Effects of three major protein sources on performance, gut morphology and fermentation characteristics in broilers. Br. Poult. Sci..

[79] Nourmohammadi R., Afzali N. (2013). Effect of citric acid and microbial phytase on small intestinal morphology in broiler chicken. Ital. J. Anim. Sci..

